# Opportune management of a patient with a macrodont and supernumerary tooth

**DOI:** 10.1111/adj.12903

**Published:** 2022-03-04

**Authors:** CYS Low, DC‐V Ong, E Freer

**Affiliations:** ^1^ School of Dentistry The University of Queensland Brisbane Queensland Australia; ^2^ Discipline of Orthodontics The University of Queensland Brisbane Queensland Australia

**Keywords:** Opportune, management, macrodont, supernumerary, tooth

## Abstract

Macrodontia is a relatively uncommon dental anomaly and has often been reported to occur in association with other dental anomalies. Significant orthodontic and restorative challenges may arise in the management of patients with macrodont teeth. This case report demonstrates the opportune and carefully considered management of a patient presenting with both a macrodont and a supernumerary incisor tooth.

## 1 INTRODUCTION

Macrodontia is a term given to teeth that are larger in dimensional size than the normal respective tooth type and have an equally enlarged pulp chamber morphology, crown and root.^1^, ^2^, ^3^ Such teeth can present in either the primary or the permanent dentition.^1^ Research estimates that the prevalence of macrodonts is around 0.03%.^4^ Macrodontia can present as an isolated or localized condition involving a single tooth in an otherwise normal dentition or a generalized condition affecting multiple teeth. Generalized macrodontia may be associated with certain medical conditions and syndromes.^5^

Macrodontia of a single tooth should not be confused with a fusion of a normal tooth with a supernumerary tooth, in which the union of two teeth results in a single large tooth.^6^ Fusion, also known as synodontia, is defined as the joining of two developing tooth germs resulting in a single large tooth structure. Fusion can occur between two normal teeth or a normal tooth and a supernumerary tooth. In contrast, the term gemination refers to the incomplete splitting of the forming dental germ into two teeth. This may present as a mesial and distal crown on a single root with a whole or divided pulpal chamber.^4^ The distinction between these two clinical presentations is often made by determining the number of teeth present,^7^ although the term double tooth is preferred if the aetiology is unknown. Incisal notching in large teeth has also been suggested as a clinical sign to assist in the differentiation between double teeth and macrodontia.^8^ The ability to distinguish between tooth fusion and gemination does not generally have any clinical consequences with respect to the overall treatment objectives.^6^

It has been hypothesized that anomalies of tooth size and number are related, thus suggesting a potentially common genetic aetiology between macrodontia and supernumerary teeth.^7^ Patients with multiple dental anomalies often require more comprehensive and considered treatment planning. The treatment of macrodont teeth can vary, depending on their clinical and radiographic presentation.^3^, ^8^

Macrodonts are very commonly asymptomatic, however, their inherently large dimensional size may negatively affect aesthetics, lead to crowding, the ectopic eruption of adjacent teeth and will likely result in unfavourable occlusion due to the disturbance of interarch tooth size relationships (i.e. causing a significant Bolton’s tooth width ratio discrepancy).^2^ Furthermore, if a supernumerary tooth is also present, extraction of a dental unit and subsequent orthodontic treatment is often required to achieve an aesthetically pleasing and functional outcome.

Supernumerary teeth may be defined as any teeth or tooth substance in excess of the usual configuration of 20 deciduous and 32 permanent teeth.^9^, ^10^, ^11^ The prevalence of supernumerary teeth in the primary dentition has been reported to range between 0.3% and 0.8%^12^ and between 0.1% and 3.8%^13^ in the permanent dentition. It appears that both genetic and environmental factors play a role in the aetiology of supernumerary teeth.^14^ Supernumerary teeth are generally classified according to their morphology or location (Table 1) and may be associated with various clinical complications (Table 2).^15^

**Table 1:** Classification of supernumerary teeth

Classification by Morphology^15^
•Conical •Tuberculate •Supplemental •Odontomes
Classification by Location^15^
•Mesiodens •Paramolar •Distomolar •Preparamolar

**Table 2:** Potential clinical complications associated with supernumerary teeth^15^

•Prevention or delay of eruption of adjacent permanent teeth •Root resorption of adjacent teeth •Displacement or rotation of adjacent permanent teeth •Intra‐arch crowding •Incomplete space closure during orthodontic treatment •Dilaceration, delayed or abnormal root development of associated permanent teeth •Complications associated with the supernumerary tooth itself •Late‐forming supernumerary teeth

The decision to leave a supernumerary tooth in situ or alternatively remove it can be controversial, with the timing of such surgical removal also up for debate.^10^, ^11^ It has been suggested that two alternatives exist.^16^ The first option involves the removal of the supernumerary tooth as soon as it has been diagnosed. This option has the potential to result in dental phobia for a young child and may even cause iatrogenic devitalization or deformation of adjacent teeth.

The second option is to leave the supernumerary tooth in situ until root development of the adjacent teeth is complete.^16^ The potential disadvantages associated with this delayed removal include loss of eruptive force of adjacent teeth, loss of space and crowding of the affected arch and possible deviation of the dental midline. Ultimately, the position, size and nature of the supernumerary tooth and the level of co‐operation of the patient will affect the overall surgical management and each case should be individually assessed.^10^ Very occasionally, a clinical scenario may exist where a supernumerary tooth can be utilized for the benefit of the patient.^1^ This case report provides an example of a rather opportune situation.

## 2 CASE REPORT

### 2.1 Clinical history and diagnosis

**Fig. 1:**
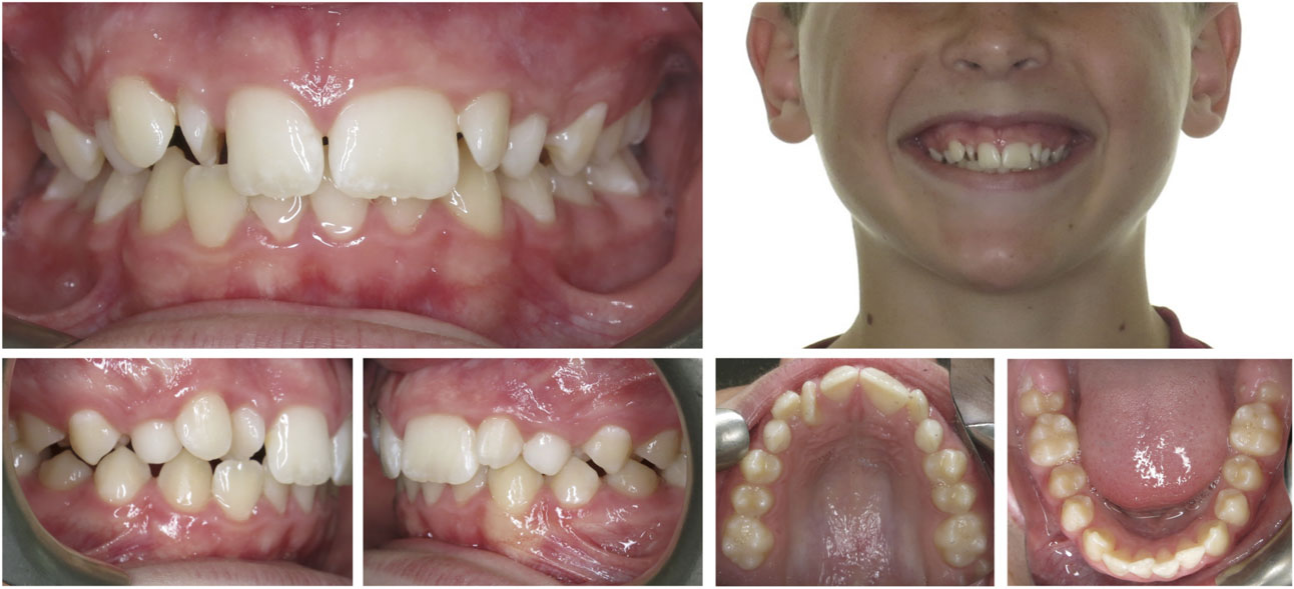
Pre‐treatment photographs of a 12‐year‐old patient with a macrodont 21. A severely rotated supernumerary permanent maxillary lateral incisor tooth was located between the 11 and 12. [Colour figure can be viewed at wileyonlinelibrary.com]

**Fig. 2:**
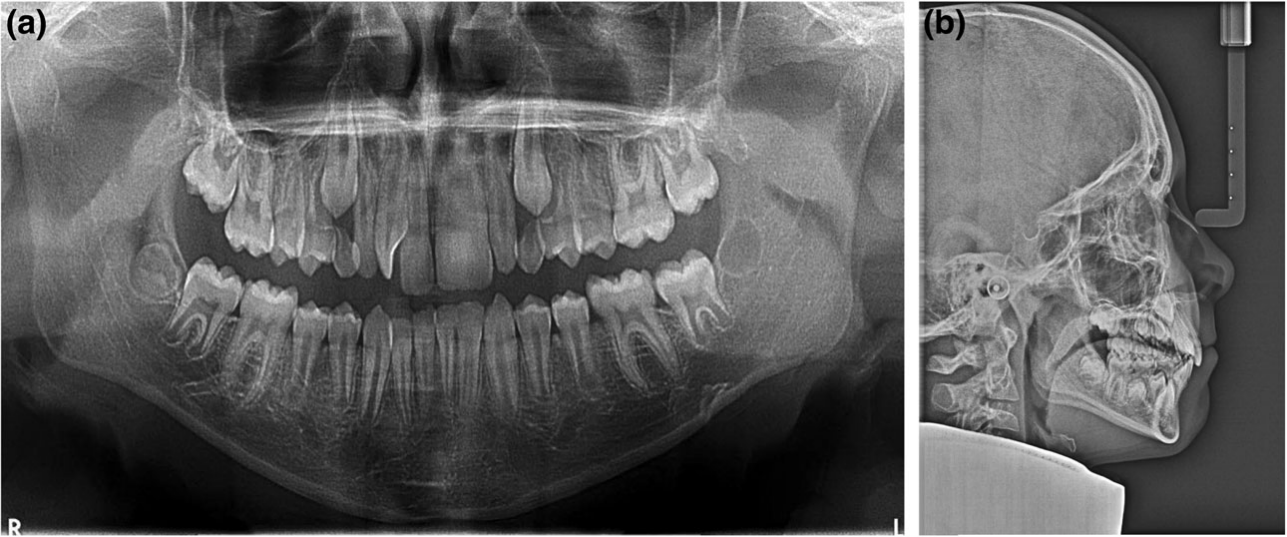
(a) The panoramic radiograph demonstrated the presence of all permanent teeth including the early crown formation of the third permanent molars. The 21 was determined to be a localized macrodont with a single large pulpal chamber. The severely rotated supernumerary permanent maxillary lateral incisor is also evident in the first quadrant (b) The lateral cephalograph revealed a mild Class II skeletal base relationship with mesofacial vertical facial proportions.

A 12‐year‐old medically healthy patient was referred for an orthodontic consultation. The presenting concern was the appearance of the maxillary anterior teeth, in particular, the oversized permanent maxillary left central incisor. This oversized tooth, presumed to be the 21, was measured to be 11 mm in mesiodistal width and the contralateral 11 tooth was 8.5 mm wide (Fig. 1). The panoramic radiograph (Fig. 2a) demonstrated that the 21 was a localized macrodont with a single large pulpal chamber and a supernumerary permanent maxillary lateral incisor in the first quadrant. Other features of note were the developing maxillary and mandibular third molars and vertical eruption paths of the permanent maxillary canine teeth (Fig. 2a).

Moderate maxillary arch crowding was noted due to the supernumerary permanent maxillary lateral incisor in the first quadrant. The severely rotated tooth located between the 11 and 12 was determined to be the supernumerary tooth (8.5 mm) as the distally positioned 12 tooth (7 mm wide) closely matched the contralateral 22 tooth in mesiodistal width (6.5 mm wide). Crowding in the mandibular arch was mild with the mandibular dental midline position located 2 mm to the right‐hand side of the maxillary dental midline position and the facial midline.

A bilateral one‐half unit Class II molar and canine relationship was evident, the anterior overjet measured to be 4 mm and the anterior vertical overbite was slightly deep, covering 50% of the crown height of the mandibular anterior teeth. The lateral cephalograph revealed a mild Class II skeletal base relationship with mesofacial vertical facial proportions (Fig. 2b).

### 2.2 Treatment objectives and treatment alternatives

The most significant problems were the exceptionally wide 21 macrodont, combined with a supernumerary permanent maxillary lateral incisor in the first quadrant, resulting in a markedly asymmetric maxillary dental arch and non‐ideal smile aesthetics. As the 21 macrodont also had an extremely wide pulp chamber, significantly reducing the mesiodistal width of this tooth was not deemed to be possible or justifiable. The supernumerary permanent maxillary lateral incisor was of good integrity and demonstrated a morphology and mesiodistal width very similar to the 11 (both teeth were 8.5 mm wide). Therefore, maintaining the supernumerary tooth and removing the macrodont was determined to be the most favourable and viable treatment option, although complex orthodontic treatment would be required.

The patient and parents were made aware that the primary aims of this ideal orthodontic treatment option were to close the large residual space incurred by removing the 21 macrodont and to utilize the supernumerary permanent maxillary lateral incisor as a replacement for the maxillary right central incisor, as the pre‐existing 11 would be moved into the 21 position. Given the potential challenges associated with this atypical orthodontic treatment plan, the informed consent discussion was very detailed. Due to the extensive translational and rotational movements required for the maxillary permanent incisor teeth, the likelihood of extended active treatment duration, the possibility of iatrogenic root resorption for the 11 were clearly outlined, along with the need for indefinite orthodontic retention. It was also mentioned that the relocated 11 may also require enamel recontouring and/or cosmetic composite restoration to more closely resemble a maxillary left central incisor in the finishing stage of active orthodontic treatment.

The major advantages of this treatment option were that the final occlusion should be balanced with pleasing smile aesthetics and, most importantly, avoid the need for any significant prosthodontic rehabilitation. Both interim and future prosthodontic rehabilitation would be required if the 21 macrodont was removed without subsequent orthodontic treatment. The diagnostic information has been summarized in Table 3. All relevant treatment options were discussed in detail, with their respective objectives, advantages and disadvantages clearly outlined (Table 4).

**Table 3:** Key diagnostic features

**Skeletal**
•Mild Class II skeletal base (Horizontal) •Mesofacial growth pattern (Vertical) •Normal facial symmetry (Transverse)
**Dental**
•½ unit Class II canine relationship (Horizontal) •Slightly increased overjet ~4 mm (Horizontal) •Slightly deep anterior overbite ~50% (Vertical) •Mandibular dental midline located 2 mm to the RHS of the maxillary dental midline and the facial midline (Transverse) •No posterior crossbites (Transverse)
**Soft tissues**
•Normal lip competency (Horizontal) •Short upper lip and increased gingival display on smiling (Vertical) •Normal chin point position (Transverse)
**Other diagnostic features of note**
•Macrodont 21 •Severely rotated supernumerary tooth located between the 11 and 12 •Slightly diminutive crown morphology of the 12 and 22 •Moderate maxillary arch crowding •Mild mandibular arch crowding •Third molar teeth present in the early stage of formation

**Table 4:** Relevant treatment options

**Treatment option 1.**
Extract the macrodont 21 in conjunction with comprehensive orthodontic fixed appliance treatment
•Extract the macrodont 21
•Relocate the 11 into the 21 position, with subsequent refinement of its root angulation and incisal edge recontouring to improve its aesthetic appearance as the maxillary left central incisor
•De‐rotate and relocate the supernumerary permanent maxillary lateral incisor to become the maxillary right central incisor
•Achieve normal anterior overjet and overbite and improved overall alignment with comprehensive orthodontic fixed appliance treatment
**Treatment option 2.**
Accept the macrodont 21 and extract the supernumerary tooth in conjunction with comprehensive orthodontic fixed appliance treatment
•Extract the severely rotated supernumerary permanent maxillary lateral incisor
•Accept the compromised aesthetics due to the asymmetric crown morphology of the 11 and 21
•Achieve normal anterior overjet and overbite and improved overall alignment with comprehensive orthodontic fixed appliance treatment
**Treatment option 3.**
Extract the macrodont 21 and the supernumerary tooth in conjunction with comprehensive orthodontic fixed appliance treatment
•Extract the macrodont 21 and the severely rotated supernumerary permanent maxillary lateral incisor
•Achieve normal anterior overjet and overbite and improved overall alignment with comprehensive orthodontic fixed appliance treatment
•Interim adhesive bridge or cobalt‐chrome partial denture following the completion of orthodontic treatment
•Placement of a restorative implant and crown in the 21 position upon skeletal maturity

### 2.3 Treatment progress

The patient exhibited good oral hygiene and was motivated to undergo orthodontic treatment. General dental examinations and preventative treatment were provided at 6 month intervals during the comprehensive orthodontic treatment. Following a carefully considered treatment planning process and obtaining appropriate informed consent, the 21 macrodont and the primary maxillary canines (53 and 63) were removed by the referring general dentist.

The comprehensive orthodontic treatment commenced in the maxillary arch initially and transitioned to include the mandibular arch following the preliminary alignment of the maxillary anterior teeth. Following the relocation of the 11 (into the 21 position), this tooth required subsequent refinement of its root angulation and incisal edge recontouring to improve its aesthetic appearance as the new maxillary left central incisor.

### 2.4 Orthodontic fixed appliance treatment

**Fig. 3:**
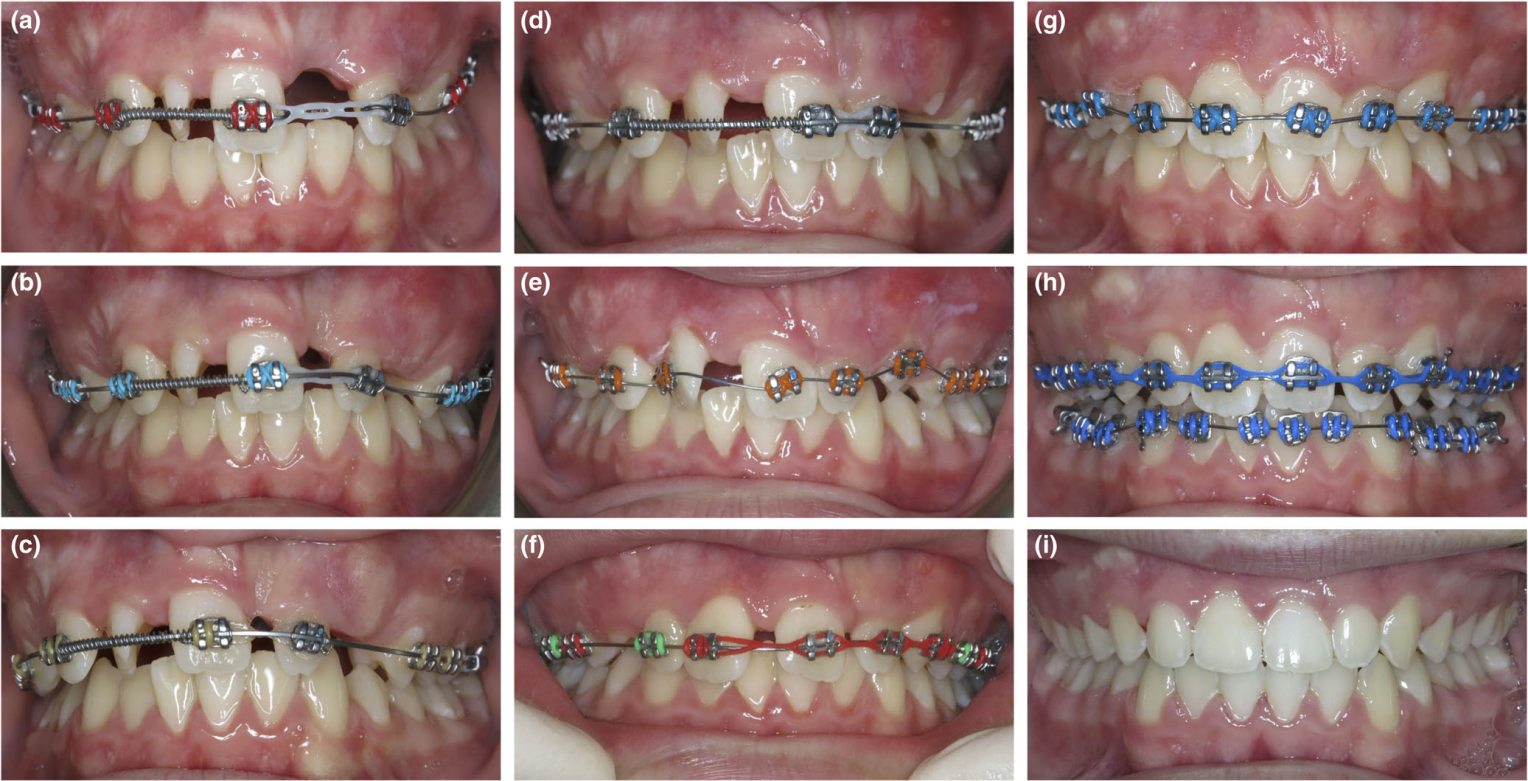
(a–i) Progress frontal photographs demonstrating the alignment and relocation of the maxillary anterior teeth (a) 1 month (b) 2 months (c) 5 months (d) 6 months (e) 10 months (f) 12 months (g) 14 months (h) 16 months (i) 27 months. [Colour figure can be viewed at wileyonlinelibrary.com]

**Fig. 4:**
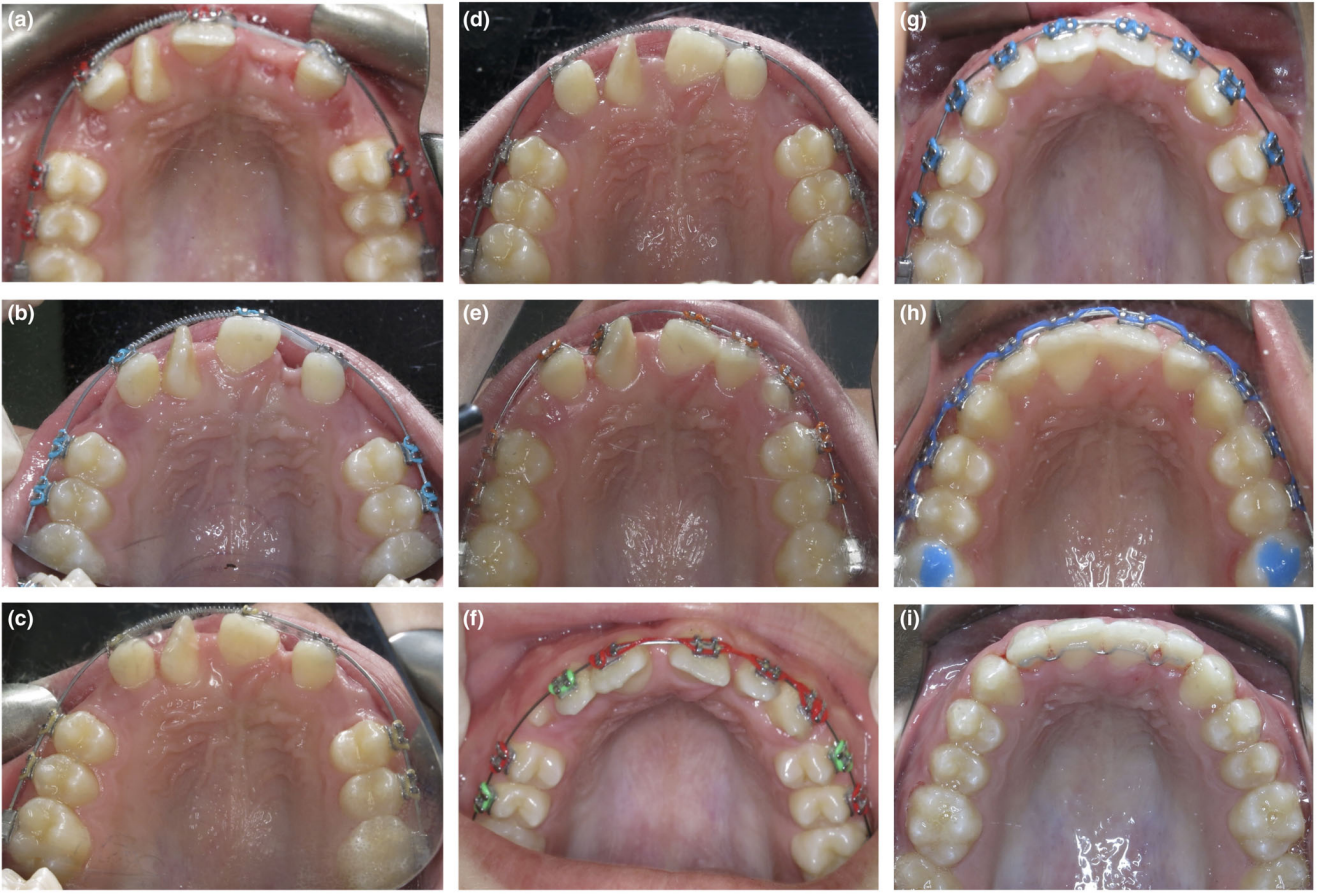
(a–i) Progress maxillary arch occlusal photographs demonstrating the alignment and relocation of the maxillary anterior teeth (a) 1 month (b) 2 months (c) 5 months (d) 6 months (e) 10 months (f) 12 months (g) 14 months (h) 16 months (i) 27 months. [Colour figure can be viewed at wileyonlinelibrary.com]

Following the extraction of the 53, 63 and 21 macrodont, pre‐adjusted maxillary labial fixed appliances (0.022” × 0.028” slot, MBT prescription, 3M Oral Care, MN, USA) were placed. Nickel‐titanium coil spring was placed on the maxillary archwire to open additional space between the 12 and 11 for the severely rotated supernumerary permanent maxillary lateral incisor. Once de‐rotated and aligned, the supernumerary permanent maxillary lateral incisor would become a replacement for the maxillary right central incisor, as the pre‐existing 11 would be moved into the 21 position. The elastomeric chain was also placed between the 11 and 22 to facilitate movement of the 11 into the 21 position (Fig. 3). Interestingly, as space was opened for the supernumerary permanent maxillary lateral incisor, this tooth appeared to de‐rotate despite no active orthodontic force being applied to it from the fixed appliances (Fig. 4). The effect of the trans‐septal fibres is the likely reason for this spontaneous de‐rotation.^18^, ^19^

At 10 months, a 11 bracket was placed on the supernumerary permanent maxillary lateral incisor. A new archwire was placed to achieve further de‐rotation and to relocate this tooth to become the maxillary right central incisor. At the same appointment, the bracket on the relocated 11 (now in the maxillary left central incisor position) was rebonded with an intentionally exaggerated 21 bracket slot angulation to further upright the root of this tooth (Fig. 3e).

At 12 months, the supernumerary permanent maxillary lateral incisor had been successfully de‐rotated and had achieved a more favourable position to resemble the maxillary right central incisor. The residual maxillary median diastema and midline position was addressed with an asymmetric elastomeric chain (Fig. 3f).

**Fig. 5:**
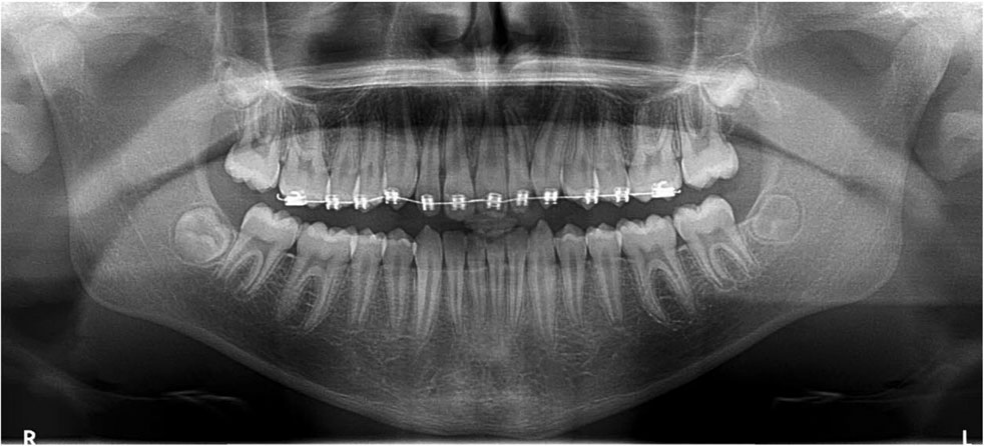
A progress panoramic radiograph taken at 14 months demonstrated pleasing root parallelism of the maxillary anterior teeth. No significant root resorption was detected on any of the repositioned maxillary anterior teeth.

At 14 months, the progress panoramic radiograph (Fig. 5) demonstrated pleasing root parallelism of the maxillary anterior teeth. Reassuringly, none of the repositioned maxillary anterior teeth demonstrated any significant root resorption. At 16 months, pre‐adjusted mandibular labial fixed appliances were placed to align the mandibular teeth and to improve the anterior overjet and overbite relationships.

**Fig. 6:**
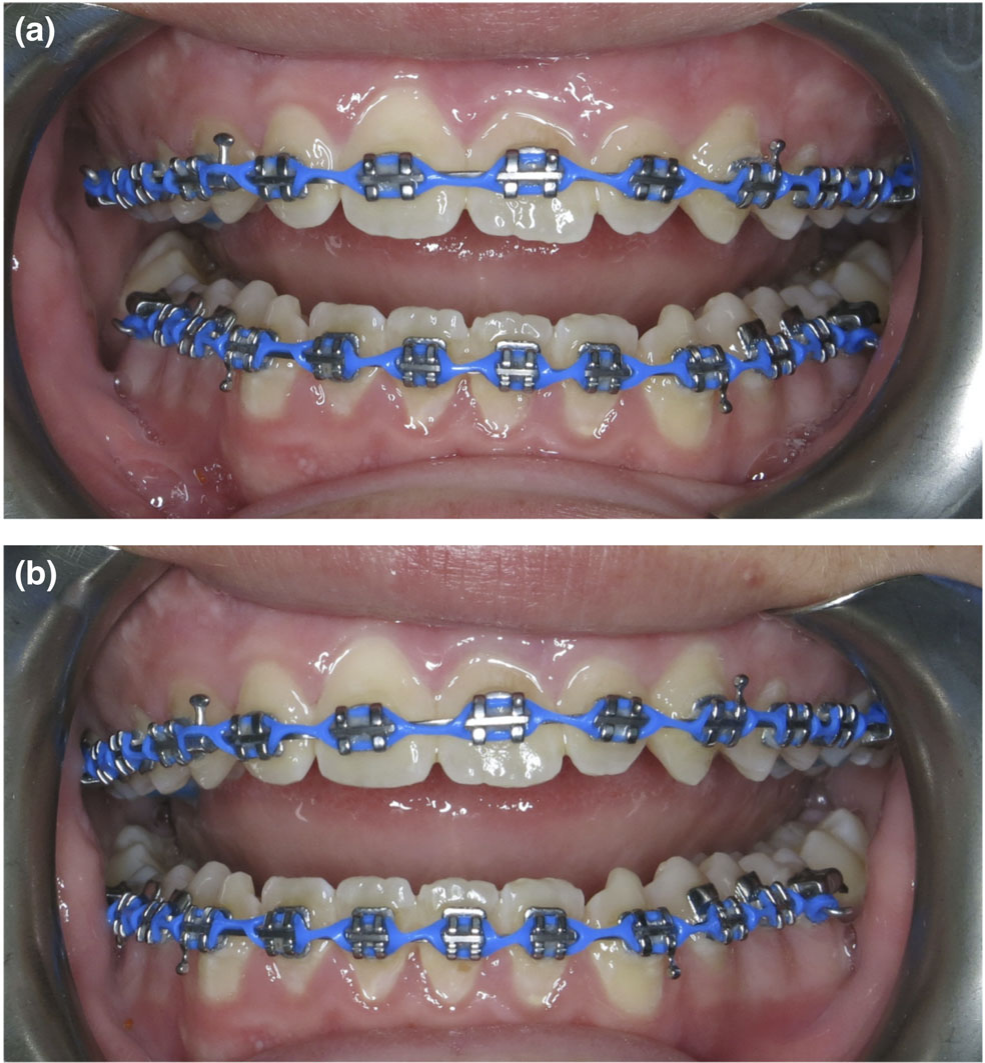
Clinical photographs taken 25 months into active treatment with an open mouth posture to better visualize the incisal edges of the maxillary anterior teeth (a) The 11 has been relocated into the maxillary left central incisor position and therefore displayed slightly aberrant incisal edge morphology. (b) This tooth was recontoured with selective and conservative enameloplasty to improve its aesthetic appearance. [Colour figure can be viewed at wileyonlinelibrary.com]

At 24 months, the relocated 11 (now in the maxillary left central incisor position) was recontoured to harmonize its morphology with the adjacent teeth (Fig. 6). The patient and family were very satisfied with the anterior smile aesthetics and did not elect to pursue any further cosmetic restorative treatment for the relocated incisor tooth.

**Fig. 7:**
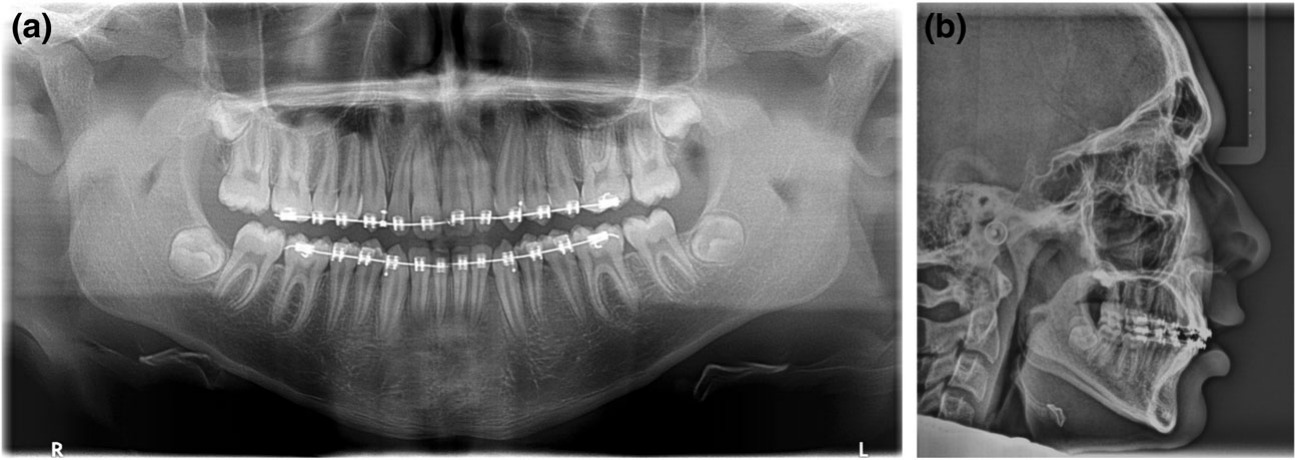
Another progress panoramic radiograph taken at 25 months was taken to evaluate the root morphology of the maxillary and mandibular teeth, along with the root parallelism and third molar development. Although the root parallelism appeared to be very satisfactory, some external apical root resorption was noted on the repositioned 11 (now located in the maxillary left central incisor position). This tooth remained asymptomatic and had no significant clinical mobility (b) The lateral cephalograph revealed acceptable incisor angulations and a normal overjet and overbite relationship.

At 25 months, another progress panoramic radiograph (Fig. 7a) was taken to evaluate the root morphology of the maxillary and mandibular teeth, along with the root parallelism and third molar development. Although the root parallelism appeared to be very satisfactory, some external apical root resorption was noted on repositioned 11 (now located in the maxillary left central incisor position). As this tooth remained asymptomatic and demonstrated no increase in mobility, the root resorption was deemed to have no appreciable clinical significance. The lateral cephalograph (Fig. 7b) revealed acceptable incisor angulations and a normal overjet and overbite relationship.

**Fig. 8:**
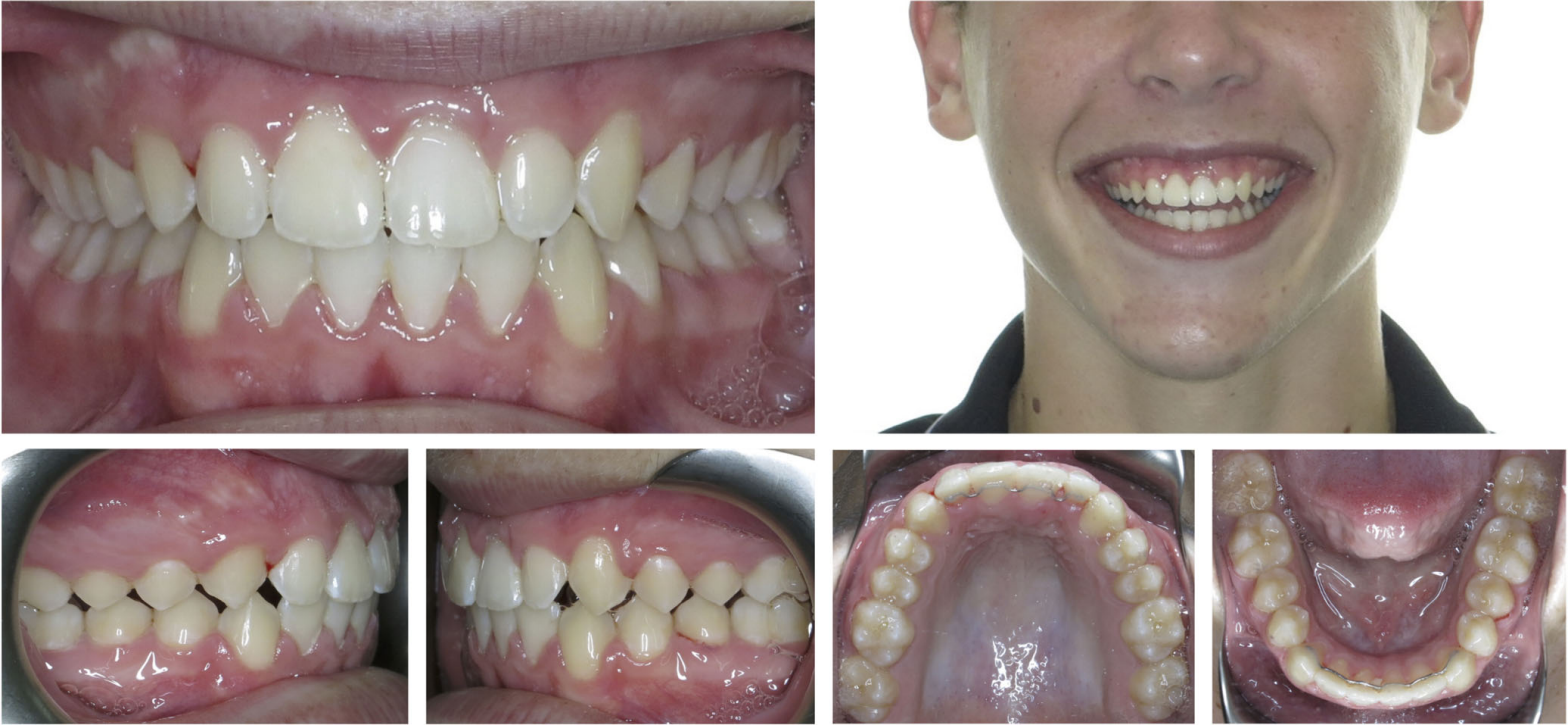
The fixed orthodontic appliances were removed after 25 months of active treatment. Maxillary and mandibular fixed retainers were placed to reduce the potential for rotational relapse of the anterior teeth. The patient and family did not proceed with any additional restorative procedures for the repositioned 11 (located in the maxillary left central incisor position). [Colour figure can be viewed at wileyonlinelibrary.com]

After 27 months, the fixed labial orthodontic appliances were removed and maxillary and mandibular fixed retainers were bonded on the anterior teeth to reduce the potential for rotational relapse (Fig. 8). Maxillary and mandibular vacuum‐formed Essix^®^ C+ material (Dentsply Raintree Essix^®^) removable retainers were also issued for indefinite nocturnal wear.

The importance of indefinite retention with fixed and removable retainers was discussed in detail prior to commencing treatment and reiterated prior to the completion of the active orthodontic treatment. Although minor changes in tooth position are expected over an individual patient’s lifetime, and generally considered to be physiologic, the supernumerary permanent maxillary lateral incisor was determined to have an inherently high potential for significant rotational relapse.

## 3 Discussion

Management of macrodont dental anomalies often proves to be very challenging. Several clinical dilemmas may become apparent, with multiple treatment options available^1^ (Table 5). Many factors (Table 6) often require careful consideration prior to selecting the final treatment option.^1^

**Table 5:** Possible treatment options for a macrodont tooth^1^

•Keep the macrodont without any further treatment or modification •Restorative adjustments for the macrodont to improve its cosmetic appearance (if such treatment does not significantly compromise the pulpal vitality of the macrodont) •Enamel reduction/stripping for the macrodont (if such treatment does not significantly compromise the pulpal vitality of the macrodont) •Endodontic treatment for the macrodont, followed by surgical hemi‐section •Extraction of the macrodont and subsequent orthodontic space closure •Extraction of the macrodont and subsequent prosthetic replacement •Extraction of the macrodont and autotransplantation of a premolar tooth into the site with appropriate subsequent restorative modification^17^

**Table 6:** Factors requiring consideration for patients with a macrodont tooth^1^

•The presence of any other associated dental anomalies (e.g. a supplemental supernumerary tooth, as described in this case report) •Age of the patient and motivation for treatment •Overall aesthetic expectations •Pulpal morphology (i.e. anomalous pulpal morphology may not be conducive to predictable endodontic treatment) •Associated features of the pre‐existing malocclusion (i.e. presence of spacing, crowding, anterior and posterior occlusial relationships and extent of dental asymmetry)

In this case report, significant orthodontic movement of the supernumerary permanent maxillary lateral incisor was required to achieve the desired outcome. However, it is important to note the challenges and potential consequences of moving teeth long distances. Carefully planned orthodontic biomechanics is required to provide efficient and effective tooth movement without unduly increasing the risk of iatrogenic problems.^20^

A rather unique iatrogenic concern has the potential to arise from the movement of the 11 across the mid‐palatal suture into the 21 position. Several important anatomical structures are located at the mid‐palatal suture. The nasopalatine nerve and the end branches of the nasopalatine artery are contained in the nasopalatine canal. The palatal orifice of the nasopalatine canal, also known as the incisive foramen, is located at the mid‐palatal suture.^21^ The incisive papilla and labial frenulum are also related to soft tissues of the mid‐palatal suture. Therefore, the movement of a tooth through the suture has the potential to affect the course of the canal and its content. Previous case reports^22^, ^23^ have shown that if the suture is mineralized, the tooth can move through uneventfully. However, if the suture is not mineralized, the mid‐palatal suture may become deviated in the same direction as that of tooth movement, as the connective tissue of the mid‐palatal suture is incorporated into the periodontal ligament.^24^

Another case report which utilized cone‐beam computed tomography (CBCT) data^24^ demonstrated that tooth movement across the mid‐palatal suture in an adolescent patient had little or no effect on the position of the incisive foramen. The tooth movement did alter the soft tissues, such as the buccal mucosa and periodontal tissues, however, the hard palate did not undergo significant change. Although no CBCT radiology was obtained for the patient described in this case report, no significant periodontal or endodontic issues were noted and the relocated 11 remained asymptomatic during and after its extensive orthodontic movement.

External apical root resorption is a potential problem associated with orthodontic tooth movement, which results in the permanent loss of the dental root structure.^25^, ^26^, ^27^, ^28^ Orthodontically induced root resorption can affect any tooth, although the maxillary central and lateral incisors are generally considered to be the most susceptible to resorption.^27^ From the available literature, it appears that increased force levels and increased active treatment duration may be associated with an increased risk of root resorption.^29^ A history of incisor trauma may also increase the risk of severe resorption.^30^, ^31^, ^32^ Therefore, careful treatment planning for each individual case and the use of sensible force levels remains of paramount importance.

Fortunately, only 2–5% of the orthodontically treated patients experience severe root resorption (defined as more than a quarter of the pre‐treatment root length of four incisor teeth)^33^ as severe loss of root structure may threaten the function and longevity of the affected teeth. For the vast majority of orthodontic patients, including for this case report, the extent of any root resorption during treatment is minor and not of any clinical significance.

When provided carefully and conservatively, enameloplasty can significantly improve dental aesthetics with minimal biological and financial costs to the patient (Table 7).^34^ In this case report, the relocated 11 (in the maxillary left central incisor position) was recontoured to harmonize its morphology with the adjacent teeth (Fig. 6). It may be possible to further enhance the gingival appearance of relocated anterior teeth through the use of a soft tissue laser^35^ or other periodontal procedures if deemed appropriate.^24^

**Table 7:** Comparison of the relevant treatment options

	Treatment option 1	Treatment option 2	Treatment option 3
Biological Risks	++ Increased risk of iatrogenic root resorption and rotational relapse potential due to the challenging tooth movements required	Minimal	+++ Future placement of a restorative implant in the critical aesthetic zone in a patient with increased gingival display
Financial Costs	++ Orthodontic treatment is required	+/Minimal Orthodontic treatment is desirable, yet still optional	+++ Orthodontic and prosthodontic treatment is required
Restorative Treatment	Minimal or not required	Minimal or not required	Extensive An interim prosthodontic appliance is required for aesthetics and space maintenance until skeletal maturity is attained

Although significant clinical challenges may be associated with orthodontic treatment, perhaps the greatest challenge of all is to maintain teeth in their corrected positions indefinitely. Teeth have a tendency to move back towards the original malocclusion as a result of periodontal, gingival, occlusal and growth‐related factors. However, tooth movement can also occur as a result of normal age‐related changes.^36^

Orthodontic alignment of severely rotated teeth can be inherently unstable^37^ and fixed orthodontic retainers can provide adjunctive support. Given the pre‐treatment rotation of this patient’s supernumerary permanent maxillary lateral incisor, a discussion focused on the need for long‐term fixed and removable retainer wear was essential. As part of the informed consent process for orthodontic treatment, patients must be fully aware of the commitment required to wear retainers for as long as they want to keep their teeth in their corrected positions.^36^

Adjunctive soft tissue or hard tissue procedures may also be performed with the aim to reduce the risk of orthodontic relapse.^38^ A supra‐crestal circumferential fiberotomy, also known as a pericision, is a periodontal soft tissue procedure to sever the dentogingival and interdental fibres around the neck of the teeth. These fibres reportedly play a role in the relapse of teeth following orthodontic correction via de‐rotation.^39^ A pericision is a relatively simple procedure performed with a scalpel under local anaesthetic and requires no periodontal dressing post‐surgery.

In contrast, interproximal reduction (IPR) is a hard tissue procedure which removes small amounts of enamel on either the mesial or distal surfaces of teeth, most commonly the maxillary or mandibular incisors. It has been suggested to help to reduce the likelihood of relapse following orthodontic treatment,^40^, ^41^however, any type of enamel reduction must be performed both conservatively and judiciously. Although it is not clear why interproximal enamel reduction reduces orthodontic relapse, it may be attributed to the flattening of interdental contacts, thereby increasing the stability between adjacent teeth.^38^

The patient described in this case report did not undergo any adjunctive soft or hard tissue procedures to theoretically enhance the orthodontic stability, as to date, there is no consensus regarding ideal retention protocols due to the paucity of high‐quality evidence.^42^ Even in a worst‐case scenario of significant rotational relapse of the maxillary anterior teeth, such relapse would not be considered to detract from the tremendous aesthetic and functional benefits gained from this orthodontic treatment. Although restorative implants are increasing in acceptance and popularity amongst both patients and clinicians, such treatment may not always represent the most ideal option in the critical aesthetic zone if a more conservative option is deemed to be reasonable. An actively growing patient often poses several management issues for future implant placement.^43^

## 4 Conclusion

This case report demonstrates how comprehensive diagnosis and carefully planned orthodontic treatment can greatly improve both the smile aesthetics and the occlusal relationship of a patient with a macrodont tooth. The long‐term restorative needs have been eliminated through the judicious removal of the unaesthetic macrodont and orthodontic relocation of the maxillary anterior teeth.

## AUTHOR CONTRIBUTION

CYSL and EF were the authors and collaborators. DO was the clinician, author and involved in data collection and analysis.

## CONFLICT OF INTEREST

None declared.
